# Investigating the relationship between quality of life and hope in family caregivers of hemodialysis patients and related factors

**DOI:** 10.1186/s12882-021-02578-6

**Published:** 2021-11-15

**Authors:** Seyedeh Azam Sajadi, Zahra Farsi, Roghayeh Akbari, Atefeh Sadeghi, Abazar Akbarzadeh Pasha

**Affiliations:** 1 Department of Nursing Management, Faculty of Nursing, Aja University of Medical Sciences, Tehran, Iran; 2 Medical-Surgical Nursing, Research and Community Health Departments, Faculty of Nursing, Aja University of Medical Sciences, Tehran, Iran; 3 Department of Nephrology, School of Medicine, Health Research Institute, Babol University of Medical Sciences, Babol, Iran; 4 Dialysis Ward, Shahid Beheshti Hospital, Babol University of Medical Sciences, Babol, Iran; 5 Clinical Research Development Center Shahid Beheshti Hospital, Babol University of Medical Sciences, Babol, IR Iran

**Keywords:** Hope, Quality of life, Family caregivers, Dialysis

## Abstract

**Background:**

Family caregivers of hemodialysis patients are the first and most crucial source of care at home. They experience many problems in the care of hemodialysis patients, which can affect their quality of life and hope, affecting the quality of care provided to patients. This study aimed to determine the relationship between quality of life and hope in family caregivers of hemodialysis patients.

**Methods:**

A cross-sectional (descriptive-analytical) study performed on 300 family caregivers in the east of Mazandaran province in Iran. Data were collected using the Family Caregiver Quality of Life (FQOL), SF8 and adult hope scale. Data analysis was performed in SPSS version 16, and a *P*-value of below 0.05 was considered statistically significant.

**Results:**

The results showed that, there was a direct and significant relationship between hope and quality of life. However, the quality of life was significantly lower in suburban residents, the unemployed, spouses, people with lower education and income levels, caregivers who cannot leave their patients alone, those living with their patients in the same house, and those taking care of male patients, compared to other participants (*P* < 0.05). Suburban residents, the unemployed, people with an insufficient level of income, and those living with their patients in the same house had significantly lower hope, compared to other subjects.

**Conclusion:**

Since an increase of hope and quality of life of caregivers can cause improved quality of patient care, it is recommended that hope-based educational programs and interventions be implemented for caregivers.

## Introduction

As a philosophy of care, family-centered care recognizes family importance as the focal point in all health care [1]. The support of family members and close people leads to improved survival, adherence to more successful treatment, and better quality of life in patients with chronic renal failure [2]. Family members of patients undergoing dialysis act as a partner in the process and are considered as the personal caregivers of patients. They are more affected by the dialysis process in hospitals compared to others, so the caregivers of dialysis patients have a much lower quality of life, compared to people in the same age and gender groups [3]. Tasks such as taking the patient to the hemodialysis center and staying with them during the process, preparing special foods, and taking patients to their physician’s office prevent family caregivers from taking care of their personal affairs and themselves [4]. Evidence shows that the caregivers of these patients deal with various problems and disorders, such as stress, depression, anxiety, lack of self-confidence, fatigue, social isolation, financial and communication limitations, poor marital adjustment, and low quality of life and sleep, which can disrupt their physical, social and emotional welfare [5–7]. The result of a study showed that though caring for hemodialysis patients threatens the caregiver’s psychological integrity, it provides the opportunity of development capabilities [8]. In addition, the direct relationship between the quality of life of the caregiver and the patient undergoing hemodialysis increases the importance of attention to caregivers’ quality of life [9].

Hope is another important concept for a healthy life. Threatening situations, loss, and changes made throughout life can affect a person’s level of hope [10]. One member’s illness influences the lives of everyone in the family, increasing their sadness and decreasing their hope [11]. There is a relationship between hope and issues such as wellbeing, quality of life, survival, problem-solving ability, and dealing with loss, tragedies, suffering, and loneliness [12]. Hope changes the experience of patients and their family members in living with a chronic disease and how to manage changes made in life [13]. Studies show that caregivers of dialysis patients have less chance to plan their activities and are anxious and afraid about their future due to difficulty in predicting the process of the disease and excessive care needs of the care recipient [14, 15]. In a research, Ebadi et al. evaluated family caregivers of patients undergoing hemodialysis, showing that the life of family caregivers of patients undergoing hemodialysis has an oscillating rhythm and a vague state of fear and hope influenced by care. These individuals experience a high level of stress and seek solutions to deal with their situation. They hope that kidney transplants will bring a better future for themselves and their patients [16]. In a research, the results showed that the higher the hope of caregivers, the lower their level of psychosocial tensions and the higher their feeling of being good [13]. Family caregivers’ sense of being hopeful directly affects patients [17]. Therefore, assessing the level of hope and quality of life of family caregivers and associated factors seems vital. Given a lack of a study on this topic in Iran, the present research aimed to determine the relationship between quality of life and hope in family caregivers of patients undergoing hemodialysis.

## Materials and methods

### Design, setting & sampling

This cross-sectional (descriptive-analytical) study was performed in four selected hemodialysis centers in east of Mazandaran Province in Iran. In this center, most patients were referred for hemodialysis three times a week. We evaluated caregivers of those who were referred to the clinic three times a week. The sample size was determined at 249 based on a similar study [18] and standard deviation of the variable of life quality at a 90% confidence interval and 0.8 accuracies using the Cochran formula. However, considering a 20% attrition rate, 300 family caregivers of patients undergoing hemodialysis were selected by convenience sampling.

$$n=\frac{{\mathrm{Z}}_{1-\frac{\upalpha}{2}}^2\ {\delta}^2}{{\mathrm{d}}^2}=\frac{1.63\times {9.89}^2}{0.8^2}=249$$

The inclusion criteria were age above 18 years, being a family member of a dialysis patient, and having at least 6 months of experience of care. But the exclusion criteria included unwillingness to cooperate with the researcher and history of crises such as mental and financial crises, serious illnesses, and deaths of loved ones in the last 6 months.

### Instruments

Data were collected by one author of the article. In this study, data were collected using demographic characteristics questionnaire, the questionnaire of family caregiver quality of life (FQOL), the short-form health survey (SF8), and the adult hope scale by Snyder. The FQOL was designed and validated by Sajadi et al in 2018. This tool includes 34 items and five dimensions of burden, conflict, positive perception of situations, self-actualization, and fear, and concern. This instrument is developed based on the international index of COSMIN (consensus-based standards for the selection of health measurement instruments) and the therapeutic care context of Iran. The items of the questionnaire are scored based on a five-point Likert scale (from completely disagree to completely agree), where a higher score indicates the higher quality of life. The scale content validity index/average for the entire instrument was 0.89 and the Cronbach’s alpha coefficient was 0.90, which indicated very good reliability and internal consistency. The interclass correlation coefficient of the scale was estimated at 0.97, which demonstrated the favorable reliability of the instrument [19]. The SF8 is a general tool to measure the quality of life. The tool includes eight items, six of which are scored based on a five-point Likert scale (from very good = 5 to very poor = 1), and the other two (items 1 and 4) are scored 1 (very low) to 6 (excellent). In addition, the score range of the tool is 8–42. The validity and reliability of the instrument have been confirmed in various studies. The reliability was confirmed at a Cronbach’s alpha of 0.89 [20]. It is worth mentioning there is a direct and significant correlation between SF8 and FQOL (*r* = 0.529, *P* < 0.001), which shows favorable convergent validity [19]. Developed by Snyder, the adult hope scale includes 12 multiple-choice items scored based on a four-point Likert scale from completely false to completely true. In this instrument, items 3, 5, 7, and 11 are trick questions and are scored zero points. The cut-off point of the scale is 30 and scores above 30 indicate higher hope. To assess the validity of the hope scale, Snyder and Lopez (2007) reported the internal consistency of agency and pathways dimension in the ranges of 0.67–0.71 and 0.63–0.8, respectively [21]. The Cronbach’s alpha of the tool was reported 0.89–0.91 in various studies [22, 23].

### Statistical analysis

Data analysis was performed in SPSS version 16 using the Kolmogorov–Smirnov test (to evaluate the normal distribution of the data), and Non -parametric tests such as Spearman’s test, Mann-Whitney U and Kruskal – Wallis due to Non- normal data distribution. A *P*-value of less than 0.05 was considered statistically significant.

### Ethical considerations

This research was approved by the ethics committee of Babol University of Medical Sciences, Babol, Iran, with the code of IR.MUBABOL.REC.1399.313. The research objectives were explained to all participants before the study and participation in the research was voluntary. In addition, written and oral informed consent was obtained from the subjects and they were ensured of confidentiality terms regarding their personal information. We also adhered to the instructions of the Declaration of Helsinki and the Committee on Publication Ethics (COPE) in the present study.

## Results

In this study, most caregivers were female (53%) and married (80.7%), and had an inadequate income level (54%). Other characteristics of the subjects are shown in Tables 1 and 2.

**Table 1:** The mean and standard deviation of individual characteristics of the participants

Variable	Mean (Standard Deviation)	Min	Max
**Age of caregiver (year)**	45.08 (14.19)	16	86
**Daily patient care hours**	12.98 (9.22)	1	24
**History of patient care (month)**	55.10 (62.25)	3	408
**Number of people participating in patient care besides the main caregiver**	2.96 (1.31)	1	5
**Age of patient (year)**	61.31 (14.54)	19	92
**Hope**	24.95 (3.82)	12	32
**Total SF8 score**	24.40 (6.75)	10	42
**Total FQOL**^a^** score**	103.21 (18.91)	68	162
**History of dialysis (month)**	47.68 (48.08)	6	336

*FQOL* Family caregiver’s quality of life^a^

**Table 2:** Relative and absolute distribution frequency based on individual variables

Variable	Categories	Absolute Frequency	Relative Frequency (%)
**Gender of caregiver**	Female	159	53
	Male	141	47
**Marital status**	Single	50	16.7
	Married	242	80.7
	Divorced	4	1.3
	Widowed	4	1.3
**Place of residents**	Urban	194	64.7
	Suburban	17	5.7
	Rural	89	29.7
**Level of education**	Illiterate or a junior-high-school diploma	128	42.7
	High-school diploma	106	35.3
	Academic degree	66	22
**Occupational status**	Housewife	129	43
	Employed	120	40
	Unemployed	16	5.3
	Retired	31	10.3
	Student	4	1.3
**Level of income**	Sufficient	27	9
	Moderate	111	37
	Insufficient	162	54
**Type of insurance**	Social security	184	61.3
	Health insurance	30	10
	Iranian health insurance	29	9.7
	Insurance of villagers	38	12.7
	Armed forces	16	5.3
	No insurance coverage	3	1
**Kinship**	Spouse	11	37
	Son	87	29
	Daughter	56	18.7
	Father	2	0.7
	Mother	10	3.3
	Sister	10	3.3
	Son or daughter-in-law	17	5.7
**Living with the patient in the same house**	Yes	228	76
	No	72	24
**Ability to leave the patient alone**	Yes	108	36
	No	101	37.3
	To a certain extent	91	30.3
**Gender of patient (Recipient of care)**	Female	146	48.7
	Male	154	51.3
**Number of people participating in patient care besides the main caregiver**	Zero	52	17.3
	One	60	20
	Two	85	28.3
	Three	55	18.3
	More than three	48	16

### Quality of life and related factors

According to the SF8 questionnaire, there was a reverse and significant correlation between age of caregiver, and number of hours of care per day with the variable of caregivers’ quality of life (*P* < 0.001). But we found a direct and significant correlation between the level of education and the quality of life of caregivers (*P* < 0.001). While, based on FQOL questionnaire, there was a direct and significant correlation was observed between the level of education and quality of life of caregivers (*P* < 0.001) (Table 3).

**Table 3:** Correlation between quality of life and hope with demographic variables

Variable	Quality of Life (SF8)		Quality of Life (FQOL)		Hope	
	^**a**^Correlation Coefficient	Level of Significance	^**a**^Correlation Coefficient	Level of Significance	^**a**^Correlation Coefficient	Level of Significance
**Age of caregiver**	−0.198	* < 0.001	−0.077	0.183	0.082	0.158
**Number of hours of care per day**	−0.225	* < 0.001	−0.111	0.056	−0.047	0.421
**History of care of the patient (month)**	0.010	0.861	0.066	0.253	0.131	*0.023
**The number of individuals participating in care besides the main caregiver**	−0.005	0.935	0.073	0.077	−0.087	0.134
**Age of patients**	0.035	0.549	0.077	0.183	0.047	0.420
**Level of education**	0.268	* < 0.001	0.218	* < 0.001	0.127	*0.028
**History of dialysis**	0.04	0.419	0.05	0.34	0.180	*0.002

*P < 0.05

^a^Spearman rho coefficient

There was no significant difference between male and female participants in terms of the quality of life (SF8) and caregivers’ quality of life (FQL). In addition, There was no significant difference between married caregivers and other subjects regarding the quality of life (SF8& FQL).

According to FQL, urban residents showed higher quality of life compared to rural and suburban areas residents (*p* < 0.05). Based on both questionnaires, people with academic education had a higher quality of life than people with non academic education (*P* < 0.05). In addition, the quality of life (SF8) in employed subjects and students were higher compared to unemployeds, retireds and housewifes (*P* < 0.05). Based on both questionnaires, subjects with sufficient income had higher quality of life (SF8 & FQL) than people with moderate and insufficient income (*P* < 0.05). There was no significant difference between subjects with various types of insurance coverage in terms of quality of life (SF8, FQL). In addition, Spouses had the lowest and brothers had the highest quality of life (SF8 & FQL) compared to offsprings, parents, sisters and sons or daughters-in-law (*P* < 0.05). According to SF8 & FQL, those who did not live in the same house with the patient had a higher quality of life than who lived in the same house (*P* < 0.05). Based on both questionnaires, subjects who could leave their patients alone for hours had a higher quality of life than who could not leave their patients alone (*P* < 0.05). Also caregivers caring for a female patient had a higher quality of life (SF8 & FQL) than caregivers caring for a male patient (*P* < 0.05) (Table 4).

**Table 4:** The difference in quality of life (SF8), caregivers’ quality of life (FQL), and hope based on individual variables

Variable	Category	Quality of Life (SF8)		Caregivers’ Quality of Life (FQOL)		Hope	
		Mean (SD)	Test and significance	Mean (SD)	Test and significance	Mean (SD)	Test and significance
**Gender of caregivers**	Female	23.61 (6.14)	^a^U = 9837 Z = −1.83 *p* = 0.06	102 (18.69)	^a^U = 10,366 Z = -1.12 *p* = 0.26	24.91 (3.77)	^a^U = 10,958 Z = −0.33 *p* = 0.73
	Male	25.30 (7.31)		104.57 (19.12)		25 (3.89)	
**Marital status**	Single	26.68 (7.80)	^b^χ^2^ = 4.61 df _=_ 3 *p* = 0.20	105.72 (18.17)	^b^χ^2^_=_ 2.79 d_f =_ 3 *p* = 0.42	24.34 (3.80)	^b^χ^2^ = 1.65 d_f =_ 3 *p* = 0.64
	Married	23.95 (6.47)		102.53 (18.81)		25.11 (3.76)	
	Divorced	22.50 (3.69)		104 (32.90)		22.50 (7.14)	
	Widowed	25 (8.40)		117.75 (19.05)		25.50 (3.87)	
**Place of residence**	Urban	24.81 (6.97)	^b^χ^2^ = 2.63 d_f =_ 2 *p* = 0.268	105.58 (19.28)	^b^χ^2^ = 14.58 d_f =_ 2 *p* = 0.001	25.32 (3.72)	^b^χ^2^ = 7.04 d_f =_ 2 *p* = 0.02
	Suburban	22.17 (4.65)		89.88 (12.04)		23.05 (4.52)	
	Rural	23.94 (6.57)		100.58 (17.86)		24.49 (3.78)	
**Level of education**	Illiterate or junior-high-school diploma	22.30 (5.63)	^b^χ^2^ = 22.17 d_f =_ 6 *p* = 0.001	98.65 (16.82)	^b^χ^2^ = 17.94 d_f =_ 6 *p* = 0.006	24.70 (3.55)	^b^χ^2^ = 8.039 d_f =_ 6 *p* = 0.235
	High-school diploma	25.25 (6.59)		103.89 (18.56)		24.76 (4.05)	
	Academic degree	27.46 (5.36)		109.69 (20.69)		25.94 (3.7)	
**Occupational status**	Housewife	23.47 (6.19)	^b^χ^2^ = 19.11 d_f =_ 8 *p* = 0.014	100.97 (18.10)	^b^χ^2^ = 11.90 d_f =_ 8 *p* = 0.156	24.83 (3.78)	^b^χ^2^ = 16.36 d_f =_ 8 *p* = 0.037
	Employed	26.22 (7.71)		104.75 (18.67)		25.05 (3.05)	
	Unemployed	21.25 (5.79)		89.87 (12.33)		23.31 (3.71)	
	Retired	23.48 (6.85)		107.09 (17.81)		25.96 (3.39)	
	Student	29.75 (5.37)		107.75 (13.91)		24 (3.16)	
**Level of income**	Sufficient	30.14 (7.71)	^b^χ^2^ = 32.41 d_f =_ 2 *p* = 0.000	113.66 (17.80)	^b^χ^2^ = 23.17 df = 2 *p* = 0.000	26.55 (3.52)	^b^χ^2^ = 11.40 d_f =_ 2 *p* = 0.003
	Moderate	25.57 (6.58)		107.06 (18.59)		25.52 (3.60)	
	Insufficient	22.64 (5.99)		98.82 (18.13)		24.29 (3.89)	
**Type of insurance**	Social security	24.80 (6.66)	^b^χ^2^ = 4.24 d_f =_ 5 *p* = 0.515	104.86 (19.30)	^b^χ^2^ = 5.46 d_f =_ 5 *p* = 0.362	25.14 (3.96)	^b^χ^2^ = 5.90 d_f =_ 5 *p* = 0.316
	Health insurance	23.26 (7.18)		97.93 (18.45)		24 (3.98)	
	Iranian health insurance	23.79 (6.85)		100.17 (17.79)		25.10 (3.38)	
	Insurance of villagers	24.68 (5.97)		101.10 (17.23)		24.42 (3.46)	
	Armed forces	24.91 (8.97)		101.87 (18.86)		25.87 (3.68)	
	No insurance coverage	21.33 (2.08)		107.33 (25.16)		23 (1)	
**Kinship**	Spouse	22.36 (6.26)	^b^χ^2^ = 19.19 d_f =_ 7 *p* = 0.008	98.97 (17.54)	^b^χ^2^ = 15.73 d_f =_ 7 *p* = 0.02	24.99 (3.36)	^b^χ^2^ = 2.73 d_f =_ 7 *p* = 0.90
	Son	25.37 (6.92)		103.98 (18.75)		24.93 (4.13)	
	Daughter	25.23 (6.84)		104.44 (19.39)		24.64 (3.86)	
	Father	28 (11.31)		101.50 (26.16)		24 (0)	
	Mother	25.70 (6.03)		104.30 (19.59)		26.30 (4.29)	
	Sister	24.30 (4.96)		108.10 (25.15)		25.40 (3.16)	
	Brother	29.42 (8.03)		111.71 (17.49)		25.28 (6.15)	
	Son or daughter-in-law	26.82 (6.52)		116 (17.25)		24.76 (4.45)	
**Living with the patient in the same house**	Yes	23.71 (6.52)	^a^U = 6104.50 Z = −3.28 *p* = 0.001	102 (19.16)	^a^U = 6921.50 Z = −2.005 *p* = 0.045	24.71 (3.59)	^a^U = 6579 Z = −2.54 *p* = 0.01
	No	26.61 (7.05)		107.04 (17.66)		25.69 (4.41)	
**Ability to leave the patient alone**	Yes	26.07 (6.90)	^b^χ^2^ = 12.120 d_f =_ 2 *p* = 0.002	105.59 (19.42)	^b^χ^2^ = 6.78 d_f =_ 2 *p* = 0.034	24.81 (3.83)	^b^χ^2^ = 2.09 d_f =_ 2 *p* = 0.351
	No	22.78 (6.62)		100.28 (20.96)		24.73 (3.72)	
	To a certain extent	24.23 (6.33)		103.62 (15.32)		25.36 (3.92)	
**Gender of patient (Recipient of care)**	Female	25.21 (6.80)	^a^U = 9677.50 Z = −2.08 *p* = 0.037	106.26 (19.36)	^a^U = 9243.50 Z = − 2.66 *p* = 0.008	25.28 (3.71)	^a^U = 10,290 Z = −1.27 *p* = 0.203
	Male	23.63 (6.64)		100.31 (18.05)		24.63 (3.90)	

^a^Mann – Whitney U Test

^b^Kruskal-Wallis Test

### Hope and related factors

There was a direct and significant correlation between the number of hours of care per month, level of education, and history of dialysis with the variable of hope (*P* < 0.05) (Table 3).

There was no significant difference between male and female participants in terms of hope. In addition, there was no significant difference between married caregivers and other subjects regarding the hope. The urban residents showed higher hope compared to rural and suburban areas residents (*p* < 0.05). But no significant difference was observed between various levels of education and hope. The level of hope in employed and retired subjects was significantly higher than housewifes and unemployed subjects (*P* < 0.05).

Also, subjects with sufficient income had hope higher than people with moderate and insufficient income (*P* < 0.05). Subjects who could leave their patients alone for hours had a hope higher than who could not leave their patients alone (*P* < 0.05). No significant difference was observed between others groups regarding hope (Table 4).

### Relationship between hope and quality of life

There was a direct and significant correlation between the score of quality of life obtained from the two instruments (SF8 & FQL) with caregivers’ hope (*P* < 0.001). In addition, there was a direct and significant correlation between caregivers’ hope and the dimensions of self-actualization, positive perception, and conflict (*P* < 0.001). But there was no correlation between caregivers’ hope and dimensions of fear, concern, and burden of care (*P* > 0.05) (Table 5).

**Table 5:** Correlation between hope and quality of life of caregivers

Variable	^**a**^Correlation Coefficient	Level of Significance
**The total score of quality of life (SF8)**	0.322	* < 0.001
**Self-actualization dimension of FQOL**	0.299	* < 0.001
**Positive perception**	0.437	* < 0.001
**Conflict**	0.239	* < 0.001
**Burden of care**	0.102	0.07
**Fear and concern**	0.111	0.055
**The total score of quality of life of dialysis patients’ caregivers (FQOL)**	0.227	* < 0.001

*P < 0.05

^a^Spearman rho coefficient

## Discussion

The present study aimed to determine the relationship between quality of life and hope in caregivers of patients undergoing hemodialysis and associated factors. According to the results, there was a direct and significant relationship between hope and quality of life, which follows the results of other studies [24, 25].

### Quality of life and related factors

The present study showed that spouses and older caregivers had lower quality of life (SF8). This may be due to spouses being usually more involved in care due to more contact with the patient. Also with increasing age, due to physical problems and reduced physical ability, the burden of care becomes more intense and the quality of life decreases. Evidence shows that female caregivers (mostly mothers and spouses) and those at higher ages had a lower quality of life [9]. In another research, there was a reverse relationship between the age of caregivers with quality of life of family caregivers of patients undergoing hemodialysis [25]. In the present study, caregivers with lower levels of education had significantly lower quality of life. This may be because a higher level of education will increase a person’s awareness and skills about improving lifestyles and problem-solving skills, and ultimately lead to an improvement in quality of life. In the study of Francisquini and Rha, there was a direct relationship between higher education and better quality of life [25, 26]. However, according to the Shdaifat study, there was no significant difference in quality of life in terms of education [18]. The reason for this inconsistency may be due to differences in the study population. In our study, there was a significant inverse relationship between the number of hours of care per day and the quality of life (SF8). As the duration of patient care increases, the burden of care increases, which can lead to a decline in quality of life. Another study that examined the quality of life and care burden of family caregivers of patients undergoing hemodialysis, showed there was a significant relationship between the number of daily hours of patient care and quality of life [27]. But in the study by Starks et al., Caregivers who cared for the patient undergoing hemodialysis for longer hours had a higher quality of life [28]. The reason for this inconsistency may be due to differences in the study population. In the present study, caregivers who could not leave their patient alone had a lower quality of life compared to others. The instability of the patient’s physical and mental condition and the patient’s high dependence on the caregiver, lead to a decrease in the caregiver’s independence in daily affairs, increase the burden of care and ultimately reduce the quality of life.

Wiedebusch et al. conducted a research to evaluate the quality of life of parents of children with chronic renal failure, reporting that one predictor of quality of life was parents’ understanding of the limitations created by their children’s disease in daily life. Parents who perceived fewer limitations caused by the illness of their children had a higher quality of life [29]. The present study showed that the quality of life did not differ significantly between different groups in terms of marriage. In the study of Shdaifat, there was no relationship between marital status and quality of life [18]. In the present study, unemployed caregivers had significantly lower quality of life (SF8). Unemployment is often associated with problems such as poverty and declining incomes, and a decline in quality of life. But in another study, there was no significant difference in the values of quality of life in terms of caregivers’ employment [18]. The reason for this inconsistency may be due to differences in the research environment, including the availability of support systems. In the present study, the quality of life (FQL) in people with lower incomes and suburban and rural residents was significantly lower than urban residents, which followed the Yihedego study [30]. Caring for a patient undergoing hemodialysis is associated with a cost to the caregiver. Also, patients undergoing hemodialysis should go to medical centers two to three times a week. Facing such problems for low-income caregivers and non-urban residents will be more due to the distance from medical centers and can lead to a decline in quality of life. In the present study, the quality of life (FQL) in caregivers who lived with the patient in the same home was significantly lower than others. Living with a patient in a home is usually associated with a greater burden of care and reduced caregiver independence, and can lead to a decline in quality of life. But in the study by Starks et al., Caregivers who lived with the patient had a significantly higher quality of life [28]. The reason for this disparity may be due to differences in the research environment, including the existence of different cultural contexts. Also, there was no significant difference in quality of life between different groups according to the patient’s gender and insurance status, based on both questionnaires. The researcher did not find a study consistent or inconsistent with this finding.

### Hope and related factors

In the present study, there was no relationship between age and hope, Meanwhile, a study revealed that younger caregivers had a lower level of hope and experienced more pressure in taking care of patients with advanced cancer [17]. The reason for this difference may be due to differences in the study population and the specific characteristics of each community. In the present study, there was a direct relationship between the level of caregiver education and hope. This may be because people with higher education are more likely to succeed and achieve their goals and have more hope for the future. In another research, there was a direct relationship between a higher level of education and hope in caregivers of patients with schizophrenia and patients on hemodialysis [25, 31]. In the present study, the level of hope in caregivers in different groups did not differ significantly in terms of caregiver and patient gender, marital status and type of insurance. In addition, a significant association was observed between gender and marital status in dialysis patients [32]. In the Lohne study, there was no relationship between gender and hope in family caregivers [17]. While in a study on dialysis patients, there was a significant relationship between gender and marriage [31]. This lack of consistency might be due to different populations evaluated in the foregoing studies. In the present study, people with insufficient income had lower hopes. Benefiting more from economic capital is associated with meeting needs faster and increasing the chances of overcoming problems and achieving success. In another study, a direct and significant relationship was found between hope and level of income [32], which is congruent with our findings. In our study, unemployed people had hopes lower than other caregivers. But in the study of Lohne et al., There was no relationship between employment and hope [17]. The reason for this disparity may be due to differences in the study population in terms of access to social support resources. In our study, there was a direct and significant relationship between patient dialysis history and hope in family caregivers. This finding may be because over time, the caregiver gains more experience and adapts more. This growth can be associated with increased hope in the caregiver. Dehbashi et al. reported an association between hope and history of dialysis [31]. In our study, caregivers who lived with the patient had significantly lower expectations, which is in line with the study by Lohne et al. [17]. This finding may be because living with a patient makes it more difficult to plan to achieve the intended goals.

In the present study, there was a direct and significant relationship between patient care hours per month and hope in family caregivers. But there was no relationship between day care hours and the number of participants in hopeful care. The level of hope in caregivers in different groups did not differ significantly in relation to the patient and daily care hours. Also, there was no significant difference in the level of hope of caregivers who could not leave their patients alone and other caregivers. The researcher did not find a study consistent or inconsistent with this finding.

### Relationship between hope and quality of life

In the current research, we found a direct and significant relationship between the self-actualization dimension (FQL) and the level of hope in caregivers. Evidence suggests that the caregivers of patients undergoing hemodialysis experience personal growth, which can eliminate the care burden and increase their flexibility in maintaining support even in hard situations for many years [2]. Results obtained by other studies have indicated a direct relationship between post-traumatic growth and hope in family caregivers of cancer patients [33, 34]. In the present study, we observed a direct and significant correlation between the dimension of positive perception and hope in caregivers. According to the results, the psychological consequences of care can be improved by having a positive perception of care conditions (FQL) [35]. In a research, hope-based programs increased positive emotions while decreasing the impact of the negative perception of care [36]. In this study, there was a direct and significant relationship between conflict (FQL) and hope in caregivers. Conflict can arise from inconsistencies between expected behaviors associated with an individual’s role [37]. While conflict is a stressful concept, it can lead to positive changes. Conflict is a natural and dynamic phenomenon. If considered as a solvable phenomenon, conflict can help to extend communications. But lack of proper management of conflict could have dire consequences for the patient, caregiver, and the entire family [38]. A decline in quality of life, an increase of care burden, depression, aggression, stress, anxiety, and irritation are consequences of conflict [39–41]. In this research, we found no significant relationship between care burden (FQL) and hope in caregivers. No correlation was reported between care pressure and hope in a study performed on caregivers of cancer patients [17]. But Utne introduced the level of hope in caregivers and care recipients as one of the important anticipators of care burden in family caregivers of cancer patients [42]. In another study, there was a relationship between increased care burden and decreased hope. Hope has a high ability to predict care burden in family caregivers of patients with dementia [36]. This lack of consistency could be due to differences in the studied populations. The two studies were performed on cancer patients and those with Alzheimer’s disease, whereas the present study was conducted on family caregivers of patients undergoing hemodialysis.

According to the results of the current research, there was no significant relationship between fear and concern (FQL) and the level of hope in caregivers. Evidence suggests that hope plays a mediating role in the relationship between perceived stress and the burden of care [36]. Williams revealed that family caregivers do their best to remain hopeful at the height of despair. However, they also experience fear and concern on the path [43]. Goldzweig reported a weak and reverse relationship between distresses and hope in caregivers [44].

## Conclusion

According to the results of the present study, there was a direct relationship between hope and the quality of life of family caregivers of patients undergoing hemodialysis. Since the improvement of hope and quality of life can be associated with more satisfaction in family caregivers, which in return enhances the quality of patient care, we suggest that hope-based programs be designed and implemented. In addition, regarding the impact of some of the individual characteristics of caregivers on their quality of life and increased hope, paying particular attention is more important in some groups. There was no certain limitation in the present study. One strength of the current research was using two instruments to measure the quality of life, one of which was domestic and specifically designed for family caregivers, and the other one was a global tool to assess the quality of life. It is recommended that further studies be performed to assess factors affecting hope and identify the impacts of nursing and psychological interventions on the improvement of hope and quality of life of caregivers of patients undergoing hemodialysis and peritoneal dialysis. Also we suggest that in future studies, quality of life and hope and related factors in family caregivers and care recipients be examined and compared simultaneously.

## Data Availability

The datasets used and analyzed during the present study are available from the corresponding author on reasonable request.

## Abbreviations

FQOL: Family caregiver quality of life
SF8: Short-form health survey
